# Conversion of flaxseed oil into biodiesel using KOH catalyst: Optimization and characterization dataset

**DOI:** 10.1016/j.dib.2020.105225

**Published:** 2020-02-04

**Authors:** Mohammed Danish, Pradeep kale, Tanweer Ahmad, Muhammad Ayoub, Belete Geremew, Samuel Adeloju

**Affiliations:** a Bioengineering Technology Section, Malaysian Institute of Chemical and Bioengineering Technology (MICET), Universiti Kuala Lumpur, Lot 1988, Kawasan Perindustrian Bandar Vendor, Taboh Naning, 78000, Alor Gajah, Melaka, Malaysia; b Department of Mechanical Engineering, JSPM's Bhivrabai Sawant Polytechnic, Wagholi, Pune, 412207, India; c Department of Chemistry, School of Mathematics and Natural Science, Copperbelt University, Kitwe, Zambia; d Centre for Biofuel and Biochemical Research (CBBR), Department of Chemical Engineering, Universiti Teknologi PETRONAS, 32610, Seri Iskandar, Perak, Malaysia; e Department of Chemistry, College of Natural and Computational Science, Madda Walabu University, Bale-Robe, Ethiopia; f Faculty of Science, Charles Sturt University, Wagga Wagga, NSW 2650, Australia

**Keywords:** Biodiesel, Flaxseed oil, Face-centered central composite design, Response surface methodology (RSM), Transesterification

## Abstract

The dataset presented here are part of the data planned to produce biodiesel from flaxseed. Biodiesel production from flaxseed oil through transesterification process using KOH as catalyst, and the operating parameters were optimized with the help of face-centered central composite design (FCCD) of response surface methodology (RSM). The operating independent variables selected such as, methanol oil ratio (4:1 to 6:1), catalyst (KOH) weight (0.40–1.0%), temperature (35 °C–65 °C), and reaction time (30 min–60 min) were optimized against biodiesel yield as response. The maximum yield (98.6%) of biodiesel from flaxseed can achieved at optimum methanol oil ratio (5.9:1), catalyst (KOH) weight (0.51%), reaction temperature (59.2 °C), and reaction time (33 min). The statistical significance of the data set was tested through the analysis of variance (ANOVA). These data were the part of the results reported in “Optimization of process variables for biodiesel production by transesterification of flaxseed oil and produced biodiesel characterizations” Renewable Energy [1].

## Abbreviations

FCCD: Face-centered central composite design
RSM: Response surface methodology
ANOVA: Analysis of variance
*Std. Dev.*: Standard deviation
*Std Err*: Standard error
DF: Degree of freedom
*Obs*: Observed
*VIF*: Variance inflation factor
*POE*: Propagation of error
*FI*: Interactive factor
*C.V*: Coefficient of variance

**Table:** Specifications Table

Subject	*Energy*
Specific subject area	*Renewable energy, sustainability and the Environment*
Type of data	Table Graph Figure
How data were acquired	Titration method was used for biodiesel yield estimation and the yield data were set in face centered cubic design of response surface methodology approach using Design-Expert 6.0.6 (Stat-Ease, Inc. Minneapolis, USA)
Data format	Raw (.dx6 file) Analyzed data
Parameters for data collection	Volume ratio of methanol/oil, catalyst (KOH) weight percent, reaction temperature, and reaction time.
Description of data collection	The biodiesel was prepared under different operating conditions, and the data were collected through titration methods for estimating the biodiesel yield.
Data source location	*Biodiesel synthesized in chemistry laboratory, college of Natural and Computational science, Madda Walabu University, Bale-Robe,* Ethiopia City/Town/Region: Bale-Robe Country: Ethiopia
Data accessibility	All data is along with this article*.*
Related research article	T. Ahmad, M. Danish, P. Kale, B. Geremew, S.B. Adeloju, M. Nizami, M. Ayoub, Optimization of process variables for biodiesel production by transesterification of flaxseed oil and produced biodiesel characterizations. Renewable Energy, 139 (2019) 1272–1280. DOI.org/10.1016/j.renene.2019.03.036

**Table:** 

**Value of the Data** • The data set reported in this article will provide researchers with better understanding of the effects of operating parameters on the yield of biodiesel production. • The four operating parameters such as, volume ratio of methanol/oil, KOH weight percent to oil, reaction temperature, and reaction time, were selected to optimize for maximum production of biodiesel. • The face-centered central composite design (FCCD) of RSM was used to obtain the optimum value of each parameters for maximum biodiesel yield. • The data describes the optimum conditions under which flaxseed oils can be converted into biodiesel with cost effective and energy saving approach.

### 1 Data

**Fig. 1:**
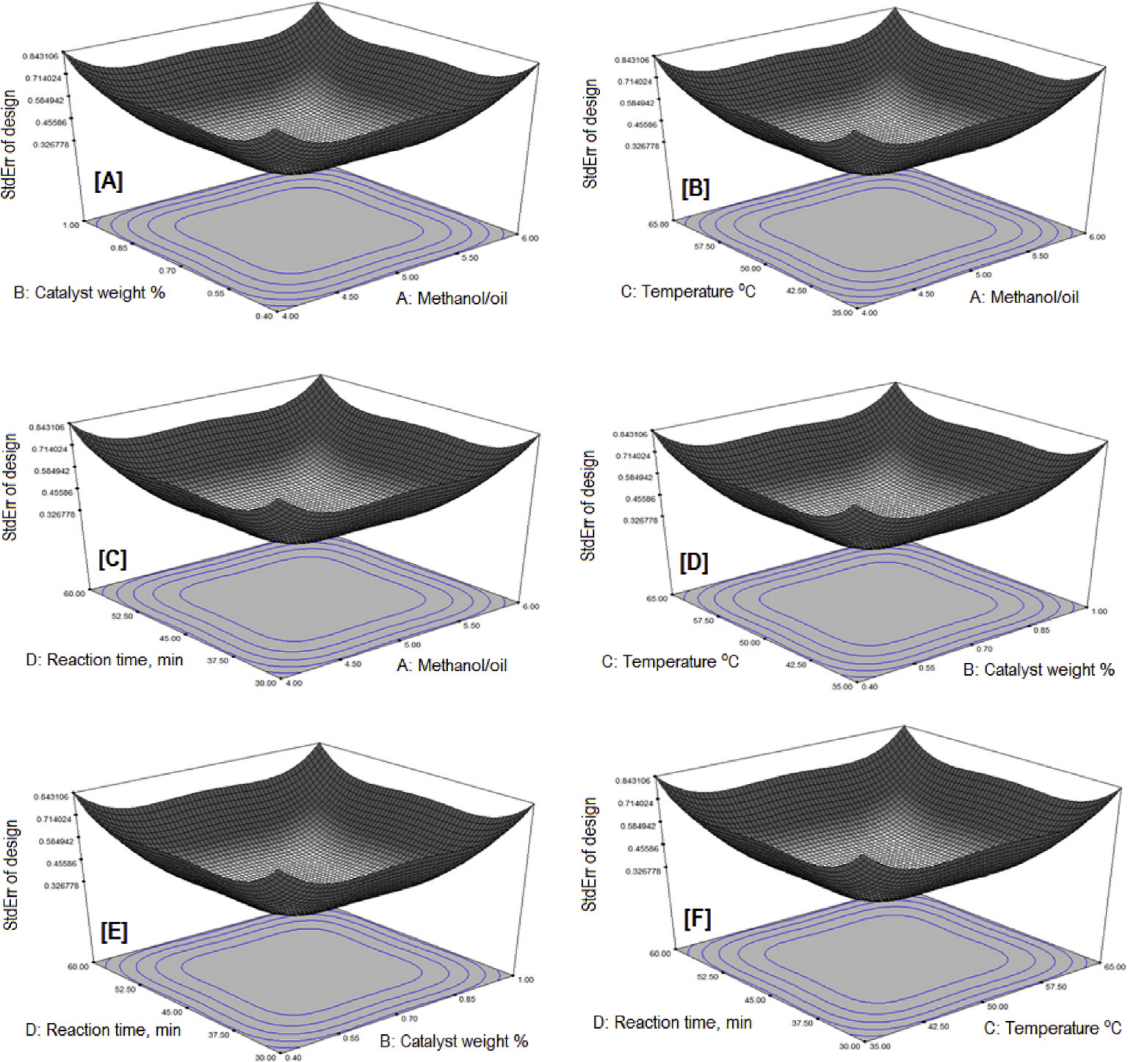
Interactive effects 3D of the process variables for the percent yield of the flaxseed biodiesel.

**Fig. 2:**
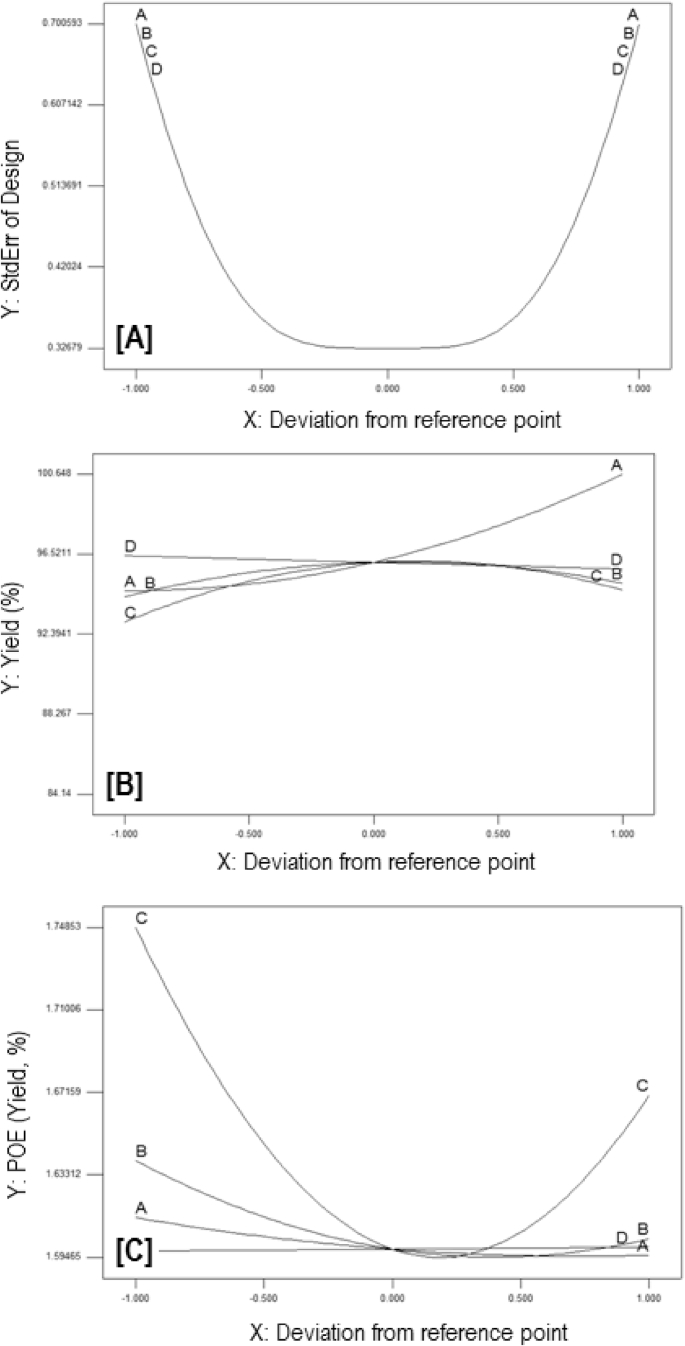
Deviation of input values of different parameter from Reference point.

**Fig. 3:**
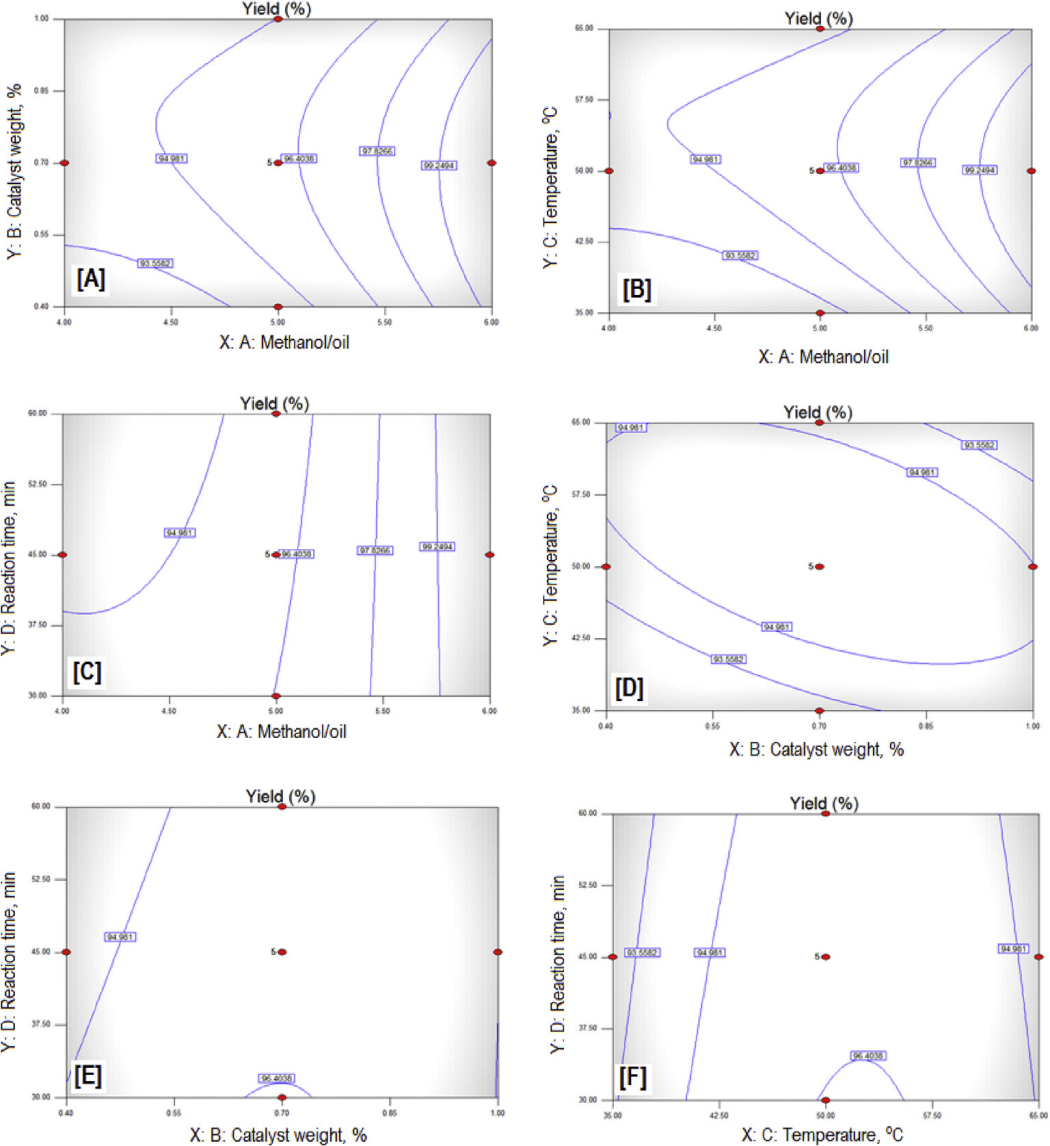
Standard Error of Design at different parameters for the percent yield of the flaxseed biodiesel.

**Fig. 4:**
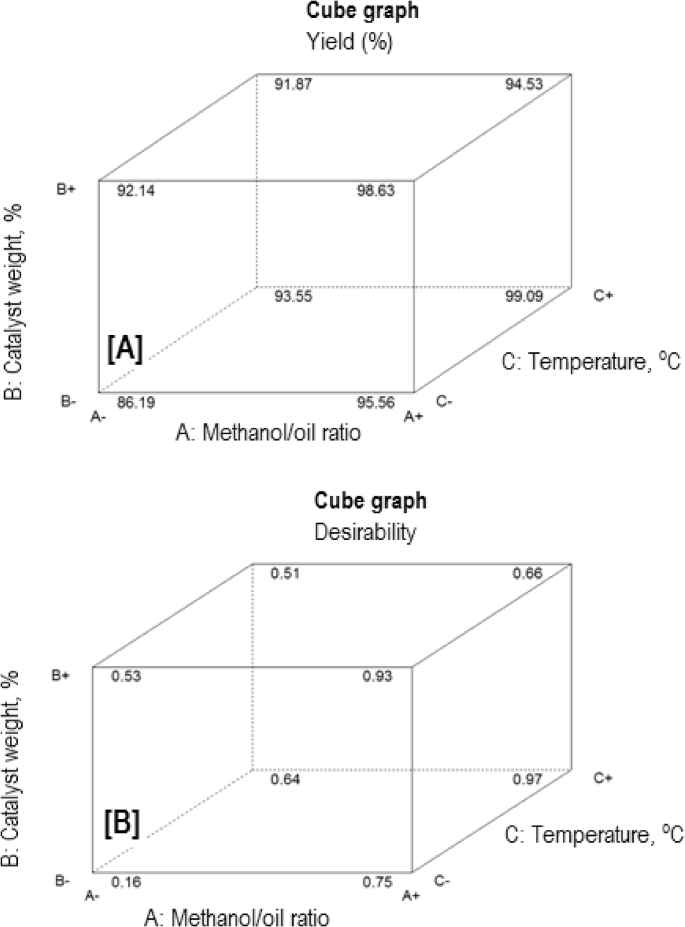
Cube graph for the maximum percent yield of the flaxseed biodiesel at different parameters.

**Fig. 5:**
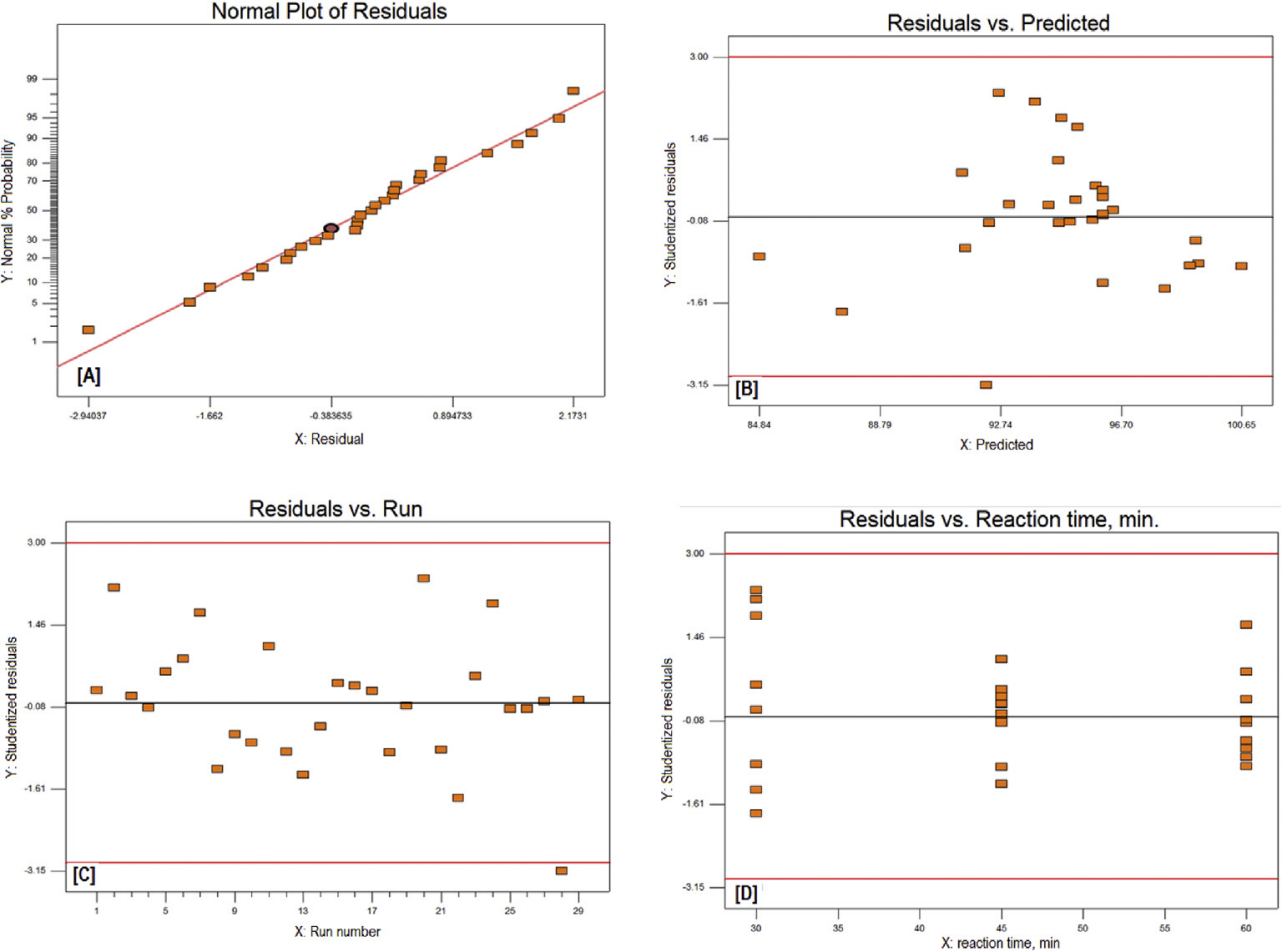
Residual variation Plots for normal and predicted value along with run and reaction time.

**Fig. 6:**
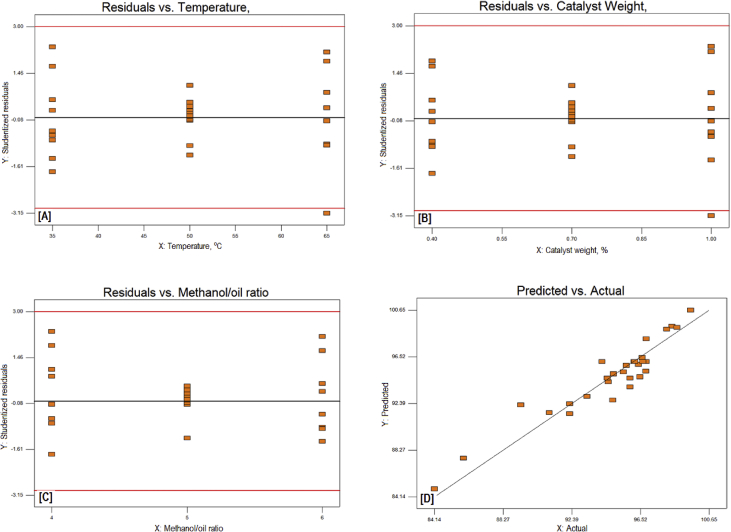
The Residual variation Plots with different process parameters.

**Fig. 7:**
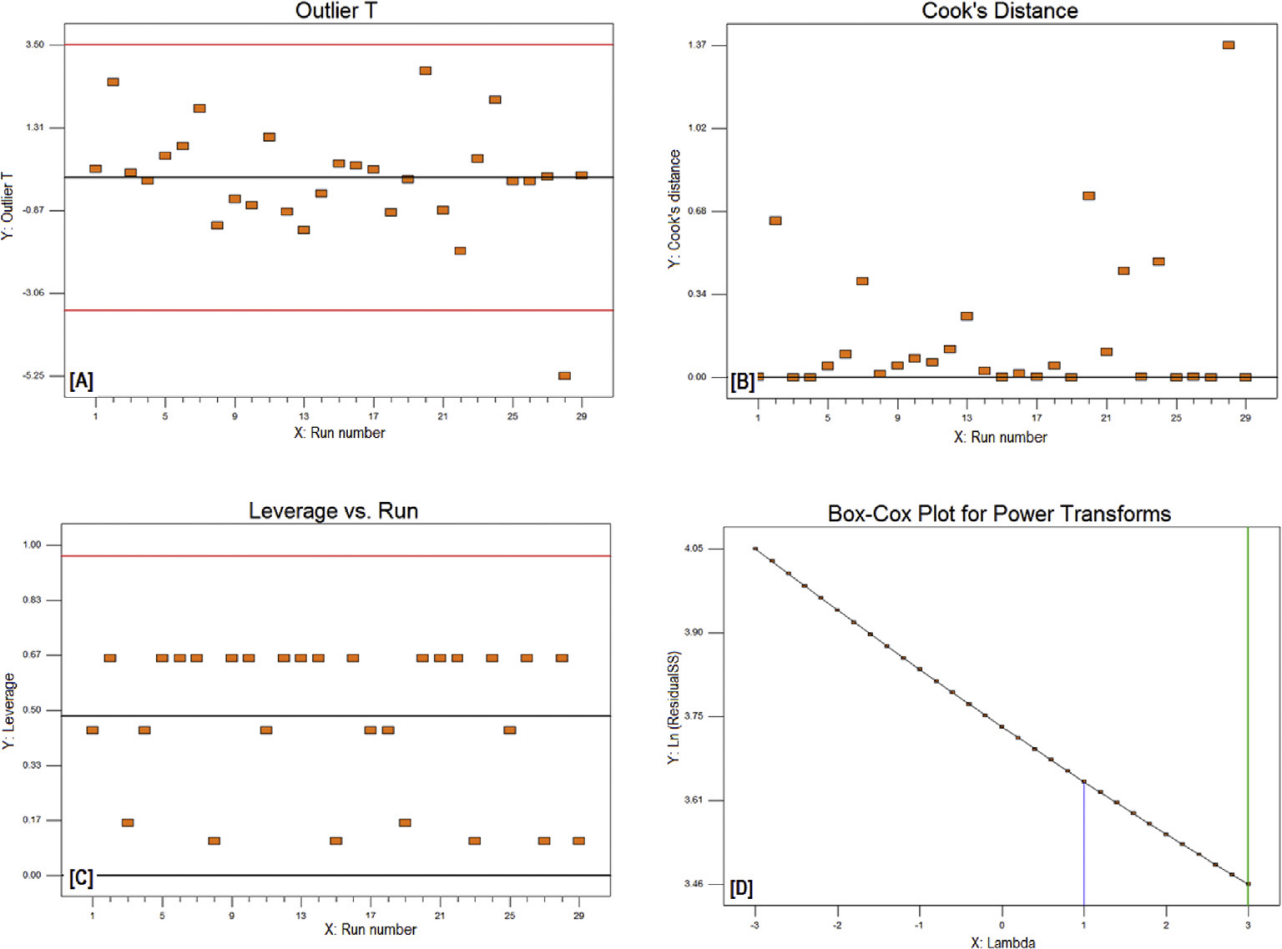
Variation in run number for Diagnostics Case Statistics.

**Fig. 8:**
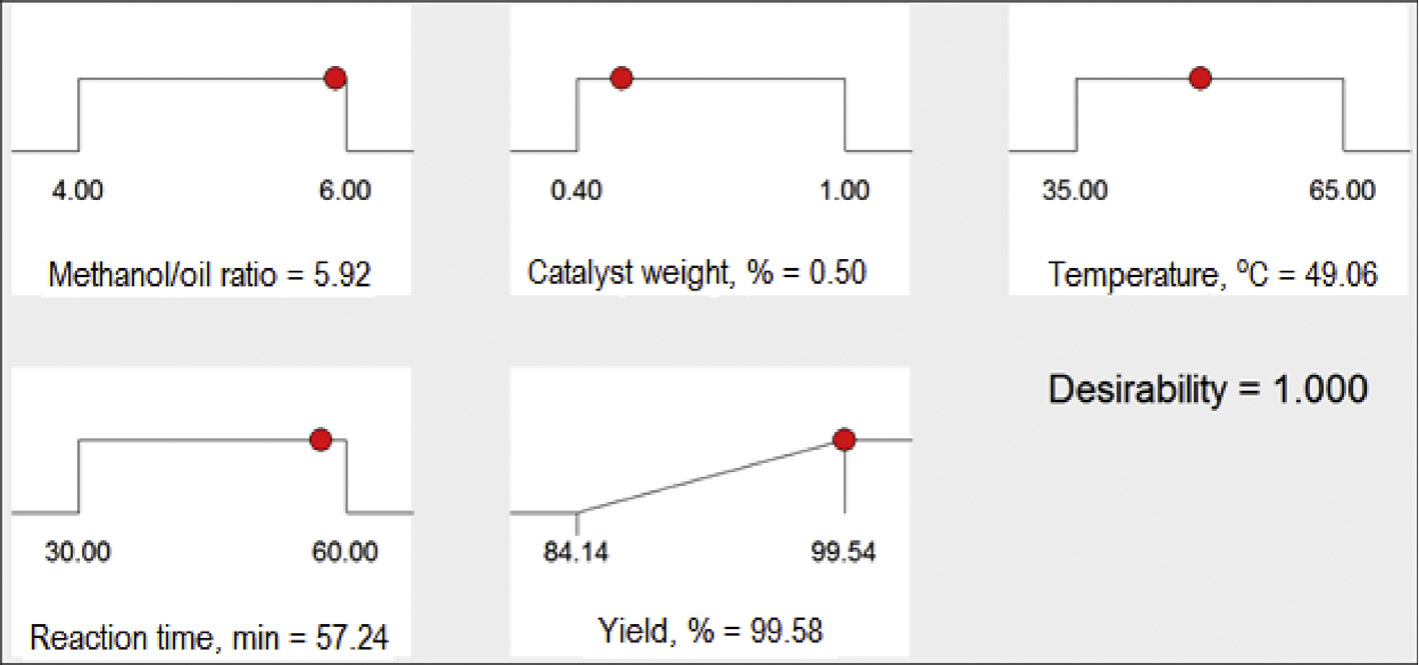
Criteria for desirability for Constraints.

The exponential growth of world population and its consequence on energy demand consumes the limited source of conventional non-renewable fossil fuel at much faster rate than expected. The rise of energy demand and fast depletion in fossil fuel triggered the research for finding the alternate source of energy. Biodiesel is one of the solutions to fulfil the energy demand as well as safety of the environment, because it is free from Sulphur, biodegradable, non-toxic, and renewable [[2], [3], [4]]. The fatty acid content of the flaxseed oil is reported elsewhere [5]. The data reported here is for the optimum production of the biodiesel from flaxseed oil. Table 1 shows the data obtained from the face-centered composite design (FCCD) approach of response surface methodology for the independent factors (methanol to oil ratio, catalyst (KOH) weight, temperature, and reaction time) and dependent factor (actual percentage yield of biodiesel) based on design of experiments. The levels and ranges of independent factors and their effect on standard deviation with measures derived from the (X’X)^−1^ matrix are elaborated in Table 2, Table 3. The parameters for prediction design and the correlation matrix of regression coefficients with correlation matrix of factors are described in Table 4. The 3D interactive effects of the process variables for the percent yield of the flaxseed biodiesel is shown in Fig. 1 while deviation of input values of different parameter from reference point depicted in Fig. 2. The sequential model sum of squares and lack of fit test and model summary statistics are discussed in Table 5, Table 6. Analysis of variance (ANOVA) table for response surface reduced quadratic model was reported elsewhere [1]. The adjustment of R-squared value parameters and coefficient estimation for final model equation along with diagnostics case statistics are illustrated in Table 7, Table 8, Table 9. Fig. 3 shows contour plot for maximum biodiesel yield within the selected independent variables (methanol to oil ratio, catalyst (KOH) weight, temperature, and reaction time) ranges. In addition, cubic graph for the maximum percent yield of the flaxseed biodiesel against independent variables and residual variation plots for normal and predicted value along with run and reaction time are shown in Fig. 4 and Fig. 5, respectively. The Residual variation plots with different process variables and variation in run number for the diagnostics case statistics are elaborated in Fig. 6, Fig. 7. The criteria for desirability for constraints is shown in Fig. 8. The point prediction and optimization of independent variables for maximum biodiesel yield from the flaxseed oil are tabulated in Table 10, Table 11 respectively.

**Table 1:** The parameter factors and actual percentage yield based on FCCD design of experiments.

Std	Run	Factor 1 A:(Methanol to oil)	Factor 2 B:(Catalyst wt.% to oil)	Factor 3 C:(Temperature) °C	Factor 4 D:(Reaction time) min	Response 1 Yield %
21	1	5 (0)	0.7 (0)	35 (−1)	45 (0)	93.30
8	2	6 (1)	1.0 (1)	65 (1)	30 (−1)	95.88
23	3	5 (0)	0.7 (0)	50 (0)	30 (−1)	96.62
20	4	5 (0)	1.0 (1)	50 (0)	45 (0)	94.90
2	5	6 (1)	0.4 (−1)	35 (−1)	30 (−1)	96.40
15	6	4 (−1)	1.0 (1)	65 (1)	60 (1)	92.26
10	7	6 (1)	0.4 (−1)	35 (−1)	60 (1)	96.84
28	8	5 (0)	0.7 (0)	50 (0)	45 (0)	94.22
11	9	4 (−1)	1.0 (1)	35 (−1)	60 (1)	91.04
9	10	4 (−1)	0.4 (−1)	35 (−1)	60 (1)	84.14
17	11	4 (−1)	0.7 (0)	50 (0)	45 (0)	95.09
14	12	6 (1)	0.4 (−1)	65 (1)	60 (1)	98.10
4	13	6 (1)	1.0 (1)	35 (−1)	30 (−1)	96.86
12	14	6 (1)	1.0 (1)	35 (−1)	60 (1)	98.72
29	15	5 (0)	0.7 (0)	50 (0)	45 (0)	96.66
16	16	6 (1)	1.0 (1)	65 (1)	60 (1)	95.50
19	17	5 (0)	0.4 (−1)	50 (0)	45 (0)	94.58
18	18	6 (1)	0.7 (0)	50 (0)	45 (0)	99.54
24	19	5 (0)	0.7 (0)	50 (0)	60 (1)	95.68
3	20	4 (−1)	1.0 (1)	35 (−1)	30 (−1)	94.86
6	21	6 (1)	0.4 (−1)	65 (1)	30 (−1)	98.41
1	22	4 (−1)	0.4 (−1)	35 (−1)	30 (−1)	85.88
27	23	5 (0)	0.7 (0)	50 (0)	45 (0)	96.86
5	24	4 (−1)	0.4 (−1)	65 (1)	30 (−1)	96.48
22	25	5 (0)	0.7 (0)	65 (1)	45 (0)	94.52
13	26	4 (−1)	0.4 (−1)	65 (1)	60 (1)	92.26
25	27	5 (0)	0.7 (0)	50 (0)	45 (0)	96.14
7	28	4 (−1)	1.0 (1)	65 (1)	30 (−1)	89.32
26	29	5 (0)	0.7 (0)	50 (0)	45 (0)	96.18

**Table 2:** Levels and ranges of independent factors used during biodiesel production from flaxseed oil.

Response	Name	Units	Obs	Minimum	Maximum	Trans	Model
Y1	Yield (%)	%	29	84.14	99.14	None	R Quadratic
Factor	Name	Units	Type	Low Actual	High Actual	Low Coded	High Coded
A	Methanol/oil ratio		Numeric	4	6	−1	1
B	Catalyst Weight %	%	Numeric	0.4	1	−1	1
C	Temperature C	0	Numeric	35	65	−1	1
D	Reaction Time min.	Min	Numeric	30	60	−1	1

**Table 3:** Power at 5% alpha level for effect of following Standard Deviation.

Term	Std Err	VIF	Ri-Squared	½ Std. Dev.	1 Std. Dev.	2 Std. Dev.
A	0.24	1	0.0	16.7%	50.6%	97.6%
B	0.24	1	0.0	16.7%	50.6%	97.6%
C	0.24	1	0.0	16.7%	50.6%	97.6%
D	0.24	1	0.0	16.7%	50.6%	97.6%
A^2^	0.62	2.64	0.6213	11.7%	32.2%	84.8%
B^2^	0.62	2.64	0.6213	11.7%	32.2%	84.8%
C^2^	0.62	2.64	0.6213	11.7%	32.2%	84.8%
D^2^	0.62	2.64	0.6213	11.7%	32.2%	84.8%
AB	0.25	1	0	15.4%	46.1%	96.0%
AC	0.25	1	0	15.4%	46.1%	96.0%
AD	0.25	1	0	15.4%	46.1%	96.0%
BC	0.25	1	0	15.4%	46.1%	96.0%
BD	0.25	1	0	15.4%	46.1%	96.0%
CD	0.25	1	0	15.4%	46.1%	96.0%

Basis std dev.=1.

**Table 4:** Parameters for prediction design.

Parameters	Value
Maximum Prediction Variance (at a design)	0.659
Average Prediction Variance	0.517
Condition Number of Coefficient Matrix	10.655
G Efficiency (calculated from the design points) (%)	78.500
Scaled D-optimality Criterion	2.510
Determinant (X’X)−^1^	1.148x10−^16^
Trace of (X’X)−^1^	2.251

**Table 5:** Sequential Model Sum of Squares and Lack of Fit test.

Source	Sum of squares	DF	Mean Square	F Value	Prob>F	
Mean	2.604x10^5^	1	2.604x10^5^			
Linear	178.93	4	44.73	6.39	0.0012	
2FI	88.30	6	14.72	3.33	0.022	
Quadratic	41.5	4	10.37	3.81	0.0268	Suggested
Cubic	26.45	8	3.31	1.70	0.2670	Aliased
Residual	11.67	6	1.94			
Total	2.604x10^5^	29	8991.48			
Linear	163.53	20	8.18	7.44	0.0322	
2FI	75.23	14	5.37	4.89	0.0683	
Quadratic	33.73	10	3.37	3.07	0.1455	Suggested
Cubic	7.27	2	3.64	3.31	0.1419	Aliased
Pure Error	4.39	4	1.10

**Table 6:** Model summary statistics.

Source	Std. Dev.	R-Squared	Adjusted R-Squared	Predicted R-Squared	Press	
Linear	2.65	0.5159	0.4352	0.1946	279.36	
2FI	2.10	0.7704	0.6429	−0.0112	350.74	
Quadratic	1.65	0.8901	0.7802	0.1915	280.43	Suggested
Cubic	1.39	0.9664	0.8430	−4.1879	1799.46	Aliased

**Table 7:** Adjustment of R-Squared value parameters.

Std. Dev.	1.59	R-Squared	0.8901
Mean	94.76	Adj R-Squared	0.7948
C.V.	1.68	Pre R-Squared	0.2014
Press	277	Adeq Precision	14.274

**Table 8:** Coefficient estimation for final model equation.

Factor	Coefficient Estimate	DF	Standard Error	95% CI Low	95% CI High	VIF
Intercept	96.10	1	0.52	95	97.19	
A	3.01	1	0.38	2.21	3.81	1
B	0.35	1	0.38	−0.45	1.15	1
C	0.82	1	0.38	0.015	1.62	1
D	−0.34	1	0.38	−1.14	0.46	1
A^2^	1.55	1	0.95	−0.47	3.57	2.41
B^2^	−1.43	1	0.95	−3.45	0.59	2.41
C^2^	−2.26	1	0.95	−4.28	−0.24	2.41
AB	−0.72	1	0.40	−1.57	0.13	1
AC	−0.96	1	0.40	−1.81	−0.11	1
AD	0.53	1	0.40	−0.32	1.38	1
BC	−1.91	1	0.40	−2.76	−1.06	1
BD	0.40	1	0.40	−0.45	1.25	1
CD	0.081	1	0.40	−0.77	0.93	1

**Table 9:** Diagnostics case statistics.

Standard Order	Actual Value	Predicted Value	Residual	Leverage	Student Residual	Cook's Distance	Outlier t	Run Order
1	85.88	87.54	−1.66	0.658	−1.784	0.437	−1.942	22
2	96.40	95.85	0.55	0.658	0.585	0.047	0.572	5
3	94.86	92.69	2.17	0.658	2.330	0.746	2.819	20
4	96.86	98.12	−1.26	0.658	−1.352	0.251	−1.393	13
5	96.48	94.74	1.74	0.658	1.861	0.476	2.050	24
6	98.41	99.22	−0.81	0.658	−0.872	0.104	−0.865	21
7	89.32	92.26	−2.94	0.658	−3.153	1.366	−5.247	28
8	95.88	93.86	2.02	0.658	2.165	0.644	2.522	2
9	84.14	84.84	−0.70	0.658	−0.747	0.077	−0.736	10
10	96.84	95.26	1.58	0.658	1.694	0.394	1.820	7
11	91.04	91.59	−0.55	0.658	−0.587	0.047	−0.574	9
12	98.72	99.13	−0.41	0.658	−0.444	0.027	−0.431	14
13	92.26	92.36	−0.10	0.658	−0.108	0.002	−0.104	26
14	98.10	98.95	−0.85	0.658	−0.913	0.114	−0.908	12
15	92.26	91.48	0.78	0.658	0.833	0.095	0.824	6
16	95.50	95.20	0.30	0.658	0.325	0.014	0.315	16
17	95.90	94.64	1.26	0.438	1.058	0.062	1.063	11
18	99.54	100.65	−1.11	0.438	−0.928	0.048	−0.923	18
19	94.58	94.31	0.27	0.438	0.222	0.003	0.215	17
20	94.90	95.01	−0.11	0.438	−0.091	0.000	−0.088	4
21	93.30	93.02	0.28	0.438	0.238	0.003	0.230	1
22	94.52	94.65	−0.13	0.438	−0.107	0.001	−0.104	25
23	96.62	96.44	0.18	0.160	0.125	0.000	0.120	3
24	95.68	95.75	−0.07	0.160	−0.050	0.000	−0.048	19
25	96.14	96.10	0.04	0.105	0.029	0.000	0.028	27
26	96.18	96.10	0.08	0.105	0.056	0.000	0.054	29
27	96.86	96.10	0.76	0.105	0.506	0.002	0.494	23
28	94.22	96.10	−1.88	0.105	−1.244	0.013	−1.269	8
29	96.66	96.10	0.56	0.105	0.374	0.001	0.363	15

Cases(s) with IOutlierTI>3.50.

**Table 10:** Point prediction for yield of the flaxseed biodiesel.

Factor	Name	Level	Low level	High Level	Std. Dev.
A	Methanol to oil ratio	5	4	6	1x10−^2^
B	Catalyst Weight	0.7	0.4	1	1x10−^2^
C	Temperature	50	35	65	2
D	Reaction Time	45	30	60	1

	Prediction	SE Mean	95% CI low	95% CI high	SE Pred	95% PI low	95% PI high
Yield (%)	96.096	0.52	95	97.19	1.68	92.52	99.67
POE (Yield (%))	1.59845

**Table 11:** Optimal processing conditions from numerical optimization.

Parameter				Yield (%)		Desirability
X_1_	X_2_	X_3_	X_4_	Predicted	Experimental	
5.90:1	0.51	59.19	32.83	99.56	98.60	1.000

### 2 Experimental design, materials, and methods

#### 2.1 Materials

The flaxseed oil was collected from the local market of Bale-Robe, Ethiopia. Methanol (CH_3_OH, 99.8% purity), sulfuric acid (H_2_SO_4_, 98%), and KOH were bought from Sigma Aldrich and were of analytical grade. During experiment 0.1 N sulfuric acid solution was used. All chemicals consumed during the biodiesel synthesis were of analytical grade.

#### 2.2 Methods

**Fig. 9:**
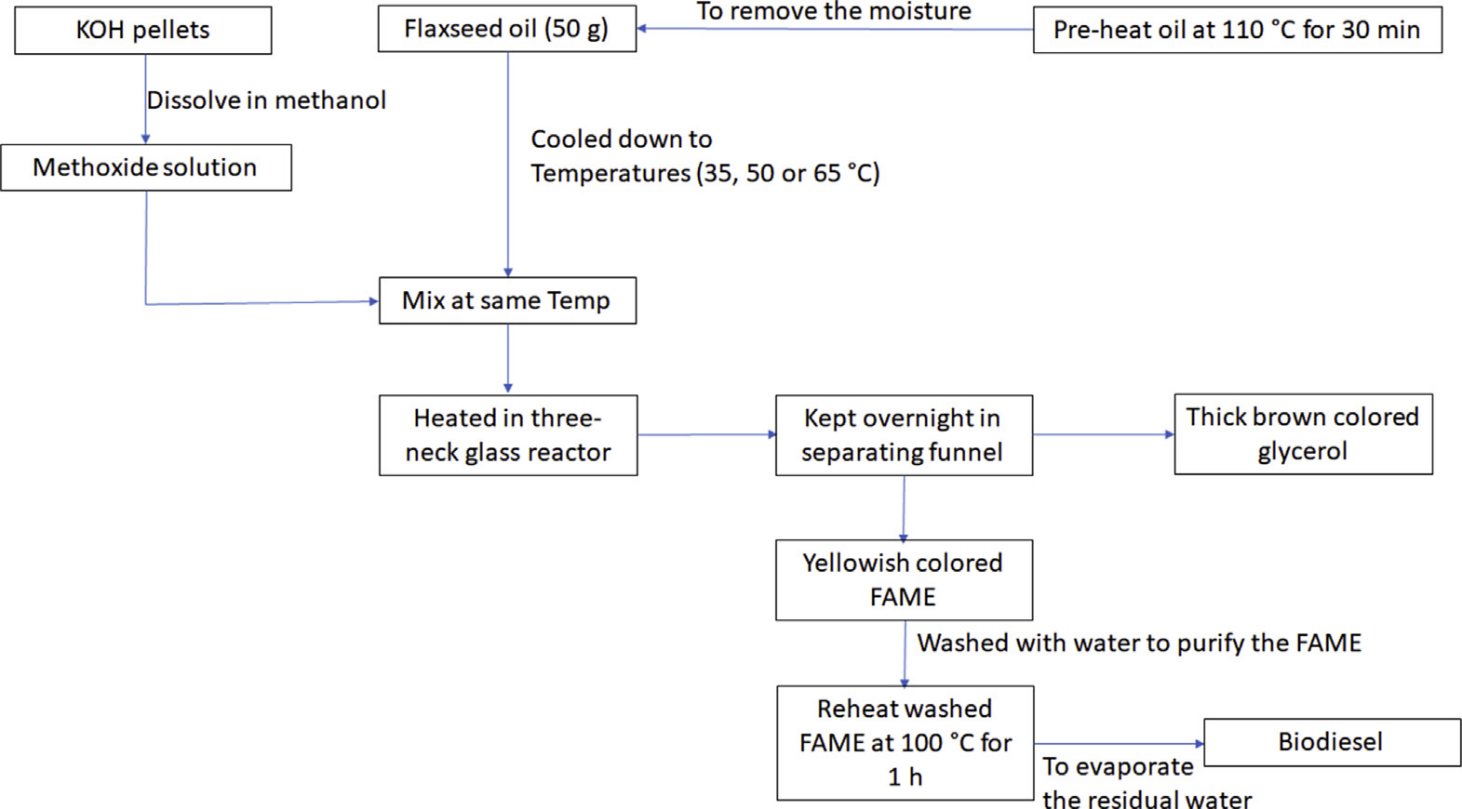
Flow chart for biodiesel production from flaxseed oil.

Biodiesel from flaxseed oil was produced in a batch experiment. The biodiesel produced in the laboratory from flaxseed oil involved a two-step transesterification reaction accompanied with product separation, washing, and drying. The process flow chart for the biodiesel production from flaxseed oil shown in Fig. 9. A fixed quantity (50 g) of the oil was measured and poured into a conical flask. The flaxseed oil was pre-heated at 110 °C for 30 min to remove the moisture content in oil. The process involves the catalyst KOH in different weight percentage of oil (0.40, 0.70, and 1.0%), methanol at various molar ratios of methanol/oil (4:1, 5:1, and 6:1) under different temperature (35, 50, and 65 °C) and reaction time (30, 45, and 60 min). The water washing method was used for further purification of FAME (biodiesel). The mixture was stirred gently to avoid foam formation. The mixture was left overnight to settle into two phases: a water-impurity phase and a biodiesel phase. Separating funnel was used to separate the FAME (biodiesel) from the water-impurity phase. This process was repeated three times to ensure the removal of most impurities from the biodiesel fraction. The washed biodiesel fraction was then reheated at 100 °C for 1 h to evaporate the residual water. The titration of biodiesel fraction with sulfuric acid (0.1 N) was used for the quantification of the FAME [6]. The percentage yield of flaxseed biodiesel was determined by comparing biodiesel weight with flaxseed oil weight used initially.

#### 2.3 Design of experiment

The face-centered central composite design (FCCD) was applied to optimize the biodiesel yield. This design is most suitable approach to optimize such processed which have a quantitative independent variable, and its response can also be observed quantitatively experimental matrix. The FCCD have sufficient tool to find the optimum values of independent variables within the selected range. Two levels and four factors with five center point values were considered for this experiment, the total number of experiments suggested through this method was (2^4^ + 2 x 4 + 5) 29 batch experiments. The independent variables selected for optimization were methanol/oil molar ratio (A), catalyst weight percent (B), reaction temperature (D) and reaction time (E). The response chosen was the biodiesel yields produced through KOH catalyzed transesterification reaction of flaxseed oil. The actual values of the independent variables are listed in Table 1. The biodiesel synthesis was conducted in batch, and each set of experimental conditions were selected randomly to minimize systematic error. All statistical parameters including analysis of variance (ANOVA) and figures were plotted with the help of Design-Expert 6.0.6 (Stat-Ease, Inc., Minneapolis, USA) [7].
